# The complete mitochondrial genome of invasive insect *Leptinotarsa decemlineata* Say 1824 (Coleoptera: Chrysomelidae)

**DOI:** 10.1080/23802359.2022.2035280

**Published:** 2022-02-07

**Authors:** Tian-Mei Dai, Hu Tian, Xu Liu, Gui-Fen Zhang, Yu-Sheng Wang

**Affiliations:** a Hunan Provincial Key Laboratory for Control of Forest Diseases and Pests, College of Forestry, Central South University of Forestry and Technology, Changsha, China; b Caofeidian Customs District P. R. China, Tangshan, China; c State Key Laboratory for Biology of Plant Diseases and Insect Pests, Institute of Plant Protection, Chinese Academy of Agricultural Sciences, Beijing, China; d Hunan Provincial Key Laboratory for Biology and Control of Plant Diseases and Insect Pests and College of Plant Protection, Hunan Agricultural University, Changsha, China

**Keywords:** *Leptinotarsa decemlineata*, Colorado potato beetle, invasive insect, phylogenetic tree, mitogenome

## Abstract

*Leptinotarsa decemlineata* Say 1824, an invasive and globally devastating beetle, inflicts great damage to potato crops worldwide. The complete mitogenome of *L. decemlineata* is described in this study. It is a 16,741 bp long circular DNA molecule with a high A + T content of 76.9%, containing a typical 37 gene pattern. All PCGs (protein-coding genes) initiate with typical ATN codons. Most PCGs use TAN as a stop codon, whereas *ND4* and *COX3* use the incomplete codon TA as the stop codon. The lengths of *rrnL* and *rrnS* genes are 1,337 bp and 811 bp, respectively. All 22 tRNAs ranged from 62 to 77 bp. Phylogenetic analysis of Chrysomelidae indicated that *L. decemlineata* clusteres with three other Chrysomelinae species, which is consistent with previous analyses.

The Colorado potato beetle, *Leptinotarsa decemlineata* Say 1824 (Coleoptera, Chrysomelidae), is one of the most destructive insect pests of potato (Cingel et al. 2016). Native to North America, it has now spread to and invaded countries worldwide (Hare 1990; Alyokhin 2009). The larvae and adults feed on foliage and tubers, causing severe damage to the plants (Alyokhin 2009). In 2017, adult specimens of *L. decemlineata* were collected from No.3 Donghuan Road, Gongliu County, Ili Kazak Autonomous Prefecture, Xinjiang Uygur Autonomous Region, China (82.3226°E, 43.4721°N). Voucher specimens were preserved in 99.7% ethanol and deposited in the Insect Collection at the College of Plant Protection, Hunan Agricultural University (please contact Yu-Sheng Wang, email: yushengwang01@163.com) under the voucher number cpb2017083101. The genomic DNA was extracted from a single specimen using the DNeasy Tissue kit (Qiagen, Germany). The mitogenome was sequenced using the Illumina Hiseq X platform (Macrogen Inc., South Korea) and assembled by SPAdes v3.11.1 (Bankevich et al. 2012). MitoZ (Meng et al. 2019) software was used to annotate the mitogenome with reference to other Chrysomelinae species (MF563962, MK049855, MF198406).

The complete mitogenome of *L. decemlineata* (GenBank accession number MZ189364) is a 16,741 bp long circular molecule, comprised 13 protein-coding genes (PCGs), 22 transfer RNA genes (tRNAs), 2 ribosomal RNA genes (*rrnL* and *rrnS*), and one control region. Nine PCGs and 14 tRNAs were encoded on the H-strand, whereas the others were located on the L-strand. The nucleotide composition of the *L. decemlineata* mitogenome is significantly biased (39.4% A, 37.5% T, 14.1% G, and 8.9% C), with an overall A + T content of 76.9%. Meanwhile, the mitogenome presented a positive AT-skew and GC-skew (0.025 and 0.226, respectively). The gene arrangement is identical with the ancestral gene order of insects (Boore 1999) and Chrysomelinae species (Gómez-Rodríguez et al. 2015), but some Chrysomelinae species have translocations of *rrnL* and *rrnS* (Nie et al. 2020). All 13 PCGs initiate with typical ATN codons: three (COX1, ND5, and ND6) with ATT, four (ATP6, COX3, ND1, and ND4) with ATG, five (COX2, CYTB, ND2, ND3, and ND4L) with ATA, and one (ATP8) with ATC. ATP6, ATP8, COX1, COX2, CYTB, ND2, ND3, ND4L, and ND6 ended by TAA as the stop codon. ND1 and ND5 ended by TAG as the stop codon. Only *ND4* and *COX3* use the incomplete codon TA as their stop codon. The *rrnL* gene is 1,337 bp long with an A + T content of 82.1%, and the *rrnS* gene is 811 bp long with an A + T content of 80.3%, as is found in most insect mitogenomes. The control region, located between the *rrnS* and *trnI* genes, is 2,109 bp long with a remarkably high A + T content (80.5%). All 22 tRNAs range from 62 bp (*trnL*) to 77 bp (*trnI*), comprising a total length of 1,435 bp. Gene overlaps are found at 21 gene junctions and account for a total length of 177 bp, ranging from 1 to 38 bp long. The longest 38 bp overlap was located between *rrnL* and *trnL1*. A total of 86 bp intergenic spacer regions are present in nine positions, ranging from 1 to 21 bp long. The largest spacer sequence of 21 bp resided between *trnL2* and *COX2*.

Phylogenetic analysis was performed with the concatenated nucleotide sequences of 13 PCGs genes from 30 Chrysomelidae species and *Spiniphilus spinicornis* (Vesperidae, as the outgroup). The 13 PCGs were partitioned using PartitionFinder2 (Lanfear et al. 2017). The phylogeny of Chrysomelidae was reconstructed using IQ-TREE (Nguyen et al. 2015) with the Maximum likelihood method. The ML phylogenetic tree supported that *L. decemlineata* clustered with three other Chrysomelinae species, and produced a Chrysomelidae phylogeny (Figure 1) which was consistent with previous analyses (Nie et al. 2020).

**Figure 1.**
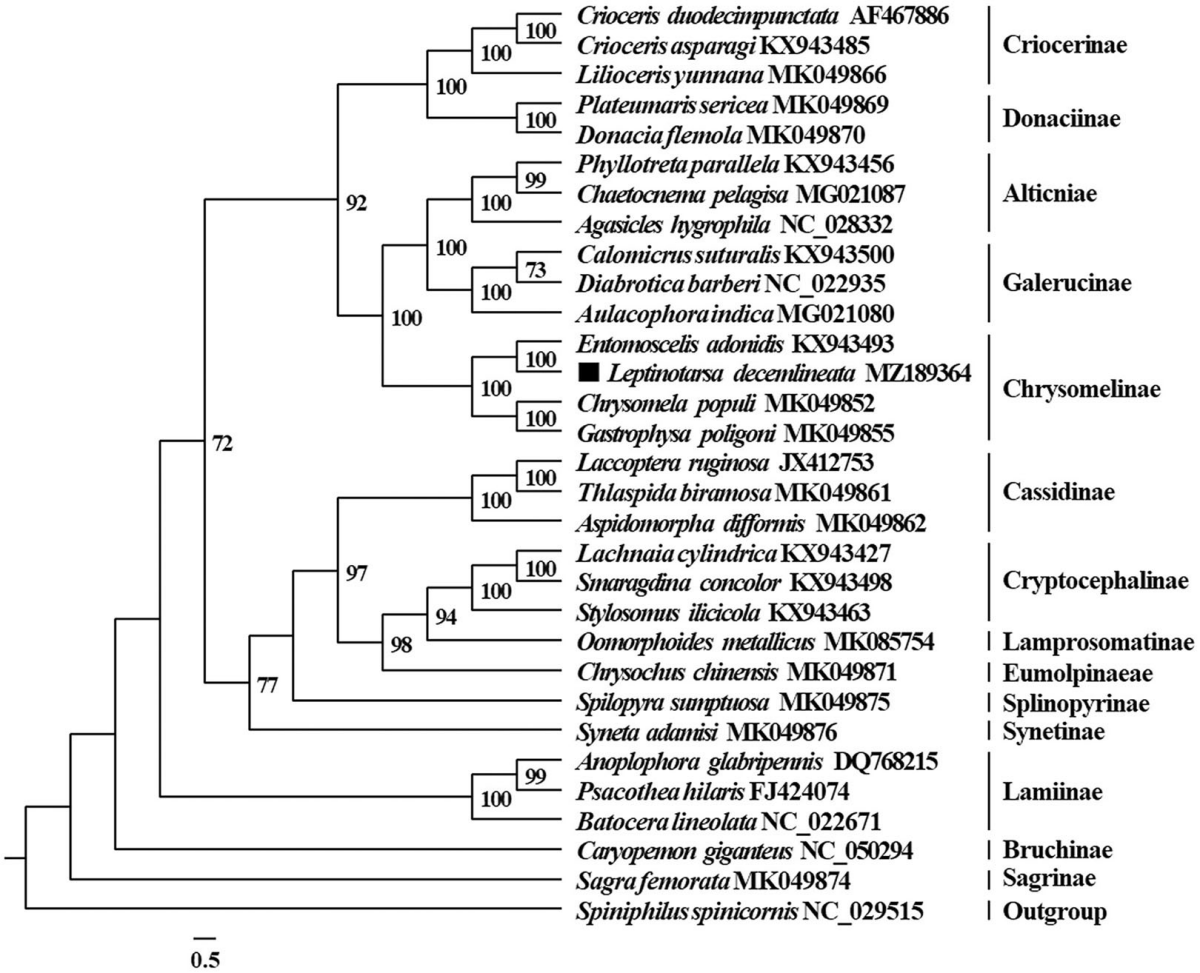
Maximum likelihood (ML) phylogenetic tree of 30 species of Chrysomelidae based on partitioned protein-coding sequences. ■ indicates the beetle determined in this study. The nodal numbers indicate the ML bootstrap support values (>70%).

## Data Availability

The genome sequence data that supports the findings of this study are openly available in GenBank of NCBI at [https://www.ncbi.nlm.nih.gov] under the accession no. MZ189364. The associated BioProject, SRA, and Bio-Sample numbers are PRJNA752722, SRR15367804, and SAMN20606172, respectively.
